# Buffalo Obstetrical Society

**Published:** 1890-12

**Authors:** 


### BUFFALO OBSTETRICAL SOCIETY.

MEETING held September 23, 1890.

Dr. C. C. FREDERICK presiding.

Dr. EUGENE A. SMITH read a paper entitled

### REPORT OF CASES OF PUERPERAL FEVER.

BY EUGENE A. SMITH, M. D.
Lecturer on Surgical Pathology, Niagara Medical College.

With only a few exceptions the medical men of the world are now agreed that the infectious disease formerly known as puerperal fever, is really puerperal septicemia, due to bacterial infection. The most sanguine and enthusiastic believers in the new pathology go so far as to declare that all post partum fever is due to bacterial infection carried to the patient by attendants or surroundings, and they hold the physician responsible whose patient develops a fever after childbirth. A smaller, more conservative class believe this opinion is too radical, and they lessen its all-embracing scope by the doctrine of auto-infection, saying a puerperal patient may contract puerperal septicemia by infection through the alimentary and respiratory tracts.

But, more than this, physicians should realize that many cases of puerperal fever cannot be grouped at all as cases of puerperal septicemia, and aside from the interest they may have, this is my object in reporting the following cases:

It may be said that the wounds of parturition are always followed by some fever during convalescence, although Case XV., a primipara, aged nineteen, had a temperature chart, showing morning and evening temperature for ten days, and the pulse ranged between seventy and eighty, and the temperature never exceeded 99°F. But Case IV., a primipara, aged twenty-one, on the sixth day had a temperature of 102°F., which fell when an enema relieved the constipation from which she suffered. Case XII., multipara, aged twenty-six, on the eleventh day had two degrees of fever, as a result of her bed being moved near a radiator. Its heat, perhaps, prevented the natural radiation of heat from her body, for upon removing her bed the ephemeral temperature fell. Case XL., primipara, aged eighteen, an unmarried mother, of slight build and neurotic, was found on the day after labor with a pulse of 120; temperature, 104½. Finding her in tears over a reproachful letter which she had received, it seemed more reasonable to ascribe the pyrexia to emotional disturbance than to sepsis, which would be exceptional upon so short an incubation. Several similar cases could be reported among the unfortunate unmarried women in the Edward-street Maternity, and the Sisters in charge have learned to recognize this ephemeral fever, which they call " Excitement or Nervous Fever." Case XLVI., primipara, aged seventeen, had right lateral and posterior laceration of the cervix after a labor antiseptically conducted. For two days she did well. On the third day, in the afternoon, without a chill, her pulse ran up to 120; temperature, 104 ; and she had cramp-like pains over the uterus and on the right side. Carbolized douches and anodynes were used. In the next three days the temperature and pulse fell, and she seemed to be normally convalescent. On the ninth day she again developed shooting pains on the right side, the temperature ran up to 104½F.; pulse, 124. Over the right ovary she could not bear the touch, and hardness from effusion was found to the right of the uterus. The cervix was nearly healed. Poultices and hot douches relieved the pain, and in two days the fever fell rapidly. Two weeks later it was noted that she felt a dragging, right-sided pelvic pain on first attempting to walk. In this case, considering the antiseptic precautions and the peculiar sharpness and short duration of the attacks, the pathological process was doubtless a pelvic cellulitis, produced by an extension of the curative inflammation from the lacerated cervix, and this inflammation terminated in resolution, no pathogenic germs having invaded the morbid territory. Pyrexia was caused by leucomaines and nervous disturbance. Mrs. M—— is a case which illustrates this point further. She is thirty-six years old, and has had six children. Her labors have been normal, with the exception of one miscarriage. There was no history of gonorrheal infection.

She was attended in her last labor, on November 10, 1889, by Dr. James S. Smith. The labor was somewhat long, but the final pains were very hard. Until November 17th the convalescence progressed normally. Then she had a chill, temperature 104°F., and pain to the touch over the uterus. In a few days the fever subsided, and on November 27th she was up and around, and was discharged. On December 9th, two weeks later, the pain returned, with temperature 101°F., and tenderness over the uterus. In a few days vaginal examination showed a tender, hardened mass behind and to the left of the uterus. From this time until April 12, 1890, the patient was confined to her bed, a condition gradually developing in the left side of the pelvis, suggesting to the touch a wooden block. At times her fever would disappear, only to return again with an extension of inflamed tissue, accompanied by bowel disturbances and hysteria. Meanwhile the patient did not fail in general condition. On December 29th, Dr. Herman Mynter saw the case with Dr. Smith, and on January 4th, January 29th, and March 9th, Dr. M. D. Mann saw her, and after careful needle exploration it was agreed no pus was present. On April 1st, after a week's use of electricity, pus was suspected from the occurrence of chill, fever and acute tenderness. Exploration followed by operation, evacuated pus which had developed below the broad ligament on the left side of the uterus, and worked to the pelvic brim, between the peritoneum and ileum. She made a rapid recovery. In this case pathogenic bacteria were presumably absent until pyogenic microörganisms appeared in the inflammatory exudate at the beginning of suppuration.

These cases of pelvic cellulitis are of a nature which makes it difficult to exclude septic infection, but they form a class which may be compared to cases of pelvic cellulitis, running the same course in the virgin, when from traumatism or operation an injury to the vagina, uterus or ovary is followed by cellular inflammation, terminating in resolution, chronicity, or suppuration.

To avoid a prolix enumeration of cases, suffice it to say that other intercurrent affections, like malaria, mastitis, hypostatic pneumonia, the exanthemata, and others could be cited as producing cases of fever after labor, which in their earlier stages were difficult to diagnose from pyrexia due to sepsis. Later, however, they were readily classed as complications of the puerperal state.

Next in this report are some undoubted cases of puerperal septicemia. The first is a case of auto-infection, occurring in the Buffalo General Hospital, during the service of Dr. C. E. Ernest. Miss ——, aged eighteen, entered, giving a history of prostitution and infection with gonorrhea several months before pregnancy. Labor was normal. High fever began at once, and in the succeeding four days she developed a severe peritonitis and died. A post mortem revealed no vaginal or uterine trouble, but a double pyosalpinx and a purulent peritonitis. One ovary and tube were found bound to the uterus by inflammatory bands, and during the contractions of labor, pus had no doubt been forced into the peritoneal cavity, thus causing the immediate onset of an unavoidable case of septic fever.

Similar cases of purulent peritonitis have occurred in the non-pregnant woman, from extension of purulent gonorrheal salpingitis. In the puerperal as well as in the unimpregnated woman, such cases are properly gonorrheal septicemia, and should be so designated, to insure accuracy of statistics in the first place, and then to relieve the physician from the censure implied in the name puerperal fever.

Cases V. and CXXXIII., primipara, were patients with vaginal diphtheria. Intra-uterine extension of the trouble and pyemia were prevented by vigorous cauterization with chloride of zinc. Both patients had fever, chills and rapid pulse, with marked abdominal pain. One had a small pelvis, and to complete the labor it was necessary to apply the Tarnier forceps above the pelvic brim. The diphtheritic process ran its course in a laceration of the perineum and vagina. A point of interest in her case was a heart murmur due to valvular lesion, remaining after an attack of rheumatism, which occurred in the fifth and sixth months of pregnancy. Her pulse before labor was 120, and intermittent. She had unusually high temperature during the sepsis; for three days between 105° and 107°F., and her pulse ranged between 140 and 160. As an internal antiseptic, sodium salicylate seemed to have an especially happy effect.

Case CXIX., primipara; aged nineteen; confined in the Lying-in-Hospital, on Edward street, had a tedious labor, the cervix descending in front of the head, and becoming edematous and thick. With much difficulty the swollen lips were pushed up and the head allowed to come down, the labor being then naturally completed. The cervix was lacerated laterally and posteriorly. This patient was sick in one room, and another patient, fifteen feet away, whom I was attending, was also in labor. In examining, I used the right hand for one and the left hand for the other. The latter patient made an uninterrupted recovery. The former developed a temperature of 104°F., and a rapid pulse on the third day. The cervix was found widely open, the uterus soft, its internal surface rough and friable. Intra-uterine douches were used every three hours. On the eighth day, the fever not abating, she had a long chill. Liquid nourishment and stimulants she took freely. She had occasional abdominal pain, with but little tympanpanitis. Antipyretics and douches did not reduce the temperature below 104°. Twice her temperature was 107°. She had two severe chills. On the seventeenth day the left knee-joint developed symptoms of arthritis, and next day the joint was aspirated, and found to contain a thin, purulent fluid. On the nineteenth day she died from exhaustion. This case was one of septic metritis, with pyemia resulting. Its origin was easy to trace. Four days before her labor another practitioner had confined a private patient in the institution. Two days after, she began having fever, and my nurse assisted in caring for her. When my patients began having labor pains before my arrival, the same nurse examined the woman who afterward had sepsis, but did not examine the other. The first septic case died on the seventh day, of purulent peritonitis, when my patient was exhibiting the first signs of her fatal malady. Before and since these cases there have been no deaths from sepsis in the institution while under my observation, a period covering two and a half years.

That much remains to be done by bacteriologists and pathologists in the classification of the septic puerperal disorders, can readily be seen from the varying etiology, symptomatology, and rational treatment of the few cases reported from my short experience. But, while all authors make some attempt at classifying the bacterial puerperal diseases, I can find no mention made of non-septic post partum fevers except in *Hirst's American System of Obstetrics.* Here part of one chapter is devoted to the "Non-infectious Fevers" of the puerperal state.

That such non-infectious fever often complicates the lying-in period, there can be no doubt, and the contribution of these cases is made in the interests of a clearer nomenclature. A differential diagnosis can be made between puerperal septicemia in its various forms and the non-infectious puerperal fevers, including traumatic cellulitis, emotional fever, constipation fever, and other ephemeral reflex febrile conditions, as well as the fever resulting from more severe intercurrent diseases.

DISCUSSION.

Dr. Marcel Hartwig: Have found the puerperal form of septic fever the most common, with and without bad odors, due to the different forms of bacteria. The gonorrheal form is another kind. I think that in time several new diseases will evolve out of puerperal fever. I have always taken the utmost care against admitting germs. I believe in auto-infection, and have had a case in which it occurred through the vagina.

Dr. W. S. Tremaine: I have clinically recognized the various forms of puerperal fever, and can see why these forms exist. The term puerperal fever should be abolished. I think that we can differentiate between ephemeral, pyogenic, pyemic and putrid forms of this fever.

Dr. DeLancey Rochester: I think it almost necessary to take the temperature before, as well as during and after confinement, in speaking about puerperal fever.

Dr. Lothrop: I think that the experience at the Maternity Hospital, where the mortality has fallen to nearly nothing, shows clearly enough the necessity of antiseptic measures before, during and after parturition.

Dr. C. C. Frederick: I have in my experience seen cases of non-septic fever in young unmarried women due to excitement. I have seen cases of high temperature without any cause, also cases in which the fever would be due to malaria, with chills and remission lasting several days.

